# Corporate social responsibility in global health: an exploratory study of multinational pharmaceutical firms

**DOI:** 10.1186/s12992-015-0100-5

**Published:** 2015-04-09

**Authors:** Hayley Droppert, Sara Bennett

**Affiliations:** Department of International Health, Johns Hopkins Bloomberg School of Public Health, 615 N. Wolfe St, Baltimore, MD 21205 USA

**Keywords:** Corporate social responsibility, Pharmaceutical firms, Global health, Emerging markets, Public-private partnerships

## Abstract

**Background:**

As pharmaceutical firms experience increasing civil society pressure to act responsibly in a changing globalized world, many are expanding and/or reforming their corporate social responsibility (CSR) strategies. We sought to understand how multinational pharmaceutical companies currently engage in CSR activities in the developing world aimed at global health impact, their motivations for doing so and how their CSR strategies are evolving.

**Methods:**

We conducted a small-scale, exploratory study combining (i) an in-depth review of publicly available data on pharmaceutical firms’ CSR with (ii) interviews of representatives from 6 firms, purposively selected, from the highest earning pharmaceutical firms worldwide.

**Results:**

Corporate social responsibility differed for each firm particularly with respect to how CSR is defined, organizational structures for managing CSR, current CSR activities, and motivations for CSR. Across the firms studied, the common CSR activities were: differential pharmaceutical pricing, strengthening developing country drug distribution infrastructure, mHealth initiatives, and targeted research and development. Primary factors that motivated CSR engagement were: reputational benefits, recruitment and employee satisfaction, better rankings in sustainability indices, entrance into new markets, long-term economic returns, and improved population health. In terms of CSR strategy, firms were at different points on a spectrum ranging from philanthropic donations to integrated systemic shared value business models.

**Conclusions:**

CSR is of increasing importance for multinational pharmaceutical firms yet understanding of the array of CSR strategies employed and their effects is nascent. Our study points to the need to (i) develop clearer and more standardized definitions of CSR in global health (2) strengthen indices to track CSR strategies and their public health effects in developing countries and (iii) undertake more country level studies that investigate how CSR engages with national health systems.

## Background

Businesses and corporations in many sectors are initiating programs and strategies aimed at enhancing social welfare, protecting the environment and defending human rights. There is evidence worldwide of the growing importance and impact of corporate social responsibility (CSR) [1]. For example, the recent United Nations (UN) Global Compact on Corporate Sustainability seeks to align objectives and interests of the business world and global community to develop innovative policies aimed at harnessing and leveraging the momentum of CSR [2]. Even more recently, the Government of India mandated CSR by requiring for-profit entities to give 2% of their net profits to charitable causes [3]. While there is extensive published literature on CSR and international development the literature on CSR and global health is limited [4,5]. A number of papers have explored whether pharmaceutical companies are living up to their human rights obligations [6-9], however, this literature falls short in considering CSR comprehensively, instead focusing narrowly on drug pricing or product licensing. Other papers on CSR in multinational pharmaceutical companies have evaluated specific CSR activities [10], focused on the creation of economic opportunity [11], or presented more of an industry perspective [12].

CSR is plagued by a multitude of definitions rooted in different sources. At its broadest, CSR has been defined as “the overall contribution of business to sustainable development” [13], characterized as economic development that does not undermine “the ability of future generations to meet their own needs” [14]. Others have envisaged it along a spectrum, which ranges from giving because it is the “right thing to do,” to risk management, all the way to creating shared value (CSV) which embeds social responsibility in the core of all corporate operations attempting to simultaneously create benefits for the company and society [15]. This study adopts the European Commission’s definition of CSR as “the responsibility of enterprises for their impacts on society.” This definition further recommends that firms put in place processes “to integrate social, environmental, ethical, human rights and consumer concerns into their business operations and core strategy in close collaboration with their stakeholders” [16]. The notion of CSR rests upon the premise that most modern firms likely create “bads” as well as goods, and accordingly should conduct activities that deliver social or environmental benefits to offset any adverse consequences of their business.

Pharmaceutical companies are special cases because their business decisions directly impact human health, making CSR efforts particularly important. These firms have been criticized for specific behaviors such as setting prohibitively high prices and sluggishness in responding to demands to provide access to life saving drugs for poor populations [12]. In response, at least in part, during the past two decades pharmaceutical companies have significantly increased CSR efforts, particularly in low- and middle-income countries (LMICs) that bear the large majority of the global disease burden [17,18]. Recent epidemiological and demographic shifts, notably the HIV/AIDS pandemic, have magnified pressures to actively work to promote societal well-being [10].

Pharmaceutical companies have been critiqued for using CSR to repair compromised reputations or to reverse public beliefs about their commercial endeavors being unethical [19]. This paper seeks to explore why and how multinational pharmaceutical companies are engaging in CSR to improve global health. Specifically, we address the following questions:

(i) What strategies and mechanisms do multinational pharmaceutical companies use for corporate social responsibility?
(ii) What motivates these companies to engage in CSR that targets population health in LMICs?
(iii) How is understanding and conceptualization of CSR in these firms evolving?

Our aim is to enhance understanding of CSR among international public health experts and promote greater discussion about how CSR can best be leveraged to improve population health around the world.

## Methods

We conducted a small scale, exploratory study of CSR in six large multinational pharmaceutical firms. The study consisted of two major components: first, interviews with representatives from select firms; and second, a review of publicly-available corporate social responsibility documents which included firm- or industry-produced documents and external reports on CSR in the firms. In addition a non-exhaustive review of secondary literature was undertaken to inform the analysis. Reviewing secondary literature helped to elucidate an understanding of corporate social responsibility, its relationship with health outcomes and the unique positioning of pharmaceutical firms in this field. This phase of work served primarily to position our findings within the existing landscape.

A review of several sources^a^ determined that in the past several years, the following 7 firms, listed in alphabetical order, were most consistently ranked among the top 10 largest multinational pharmaceutical companies: GlaxoSmithKline, Johnson and Johnson (Janssen), Merck & Co., Novartis, Pfizer, Roche, and Sanofi. All of these firms have active corporate social responsibility projects related to health in LMICs, an important criteria for inclusion. All of these firms employed CSR in order to integrate social concerns into their corporate strategy in LMIC contexts, as opposed to simply providing philanthropic donations unconnected to commercial strategy.

To set up interviews, firms were contacted using publicly available information or through existing contacts that we had with persons working in this field. Of all firms contacted, only Sanofi refused to be interviewed and was subsequently excluded from the analysis. All initial contacts were made through email in Winter 2014 and 6 interviews with 7 individuals were carried out in Spring 2014. The respondent provided for the interview was at the firm’s discretion. One firm provided two respondents, who were interviewed together. See the List of Respondents’ Titles section below where positions are presented in random order to preserve anonymity. The interviews were semi-structured and responses were documented with audio recordings and written notes. The audio-recordings were not transcribed but were referred back to during analysis to supplement notes and provide exact quotes. Interviewees were offered an informed consent form, which guaranteed the masking of firms’ identities in reference to their responses and ability to opt out of any questions they were not comfortable answering. All signed the consent form and agreed to have the interview recorded. Respondents were all asked the same general questions, with slightly different follow-up inquiries where appropriate. Respondents were asked specifically about CSR practices given their current roles but responses were likely informed by prior experiences as well. This study underwent ethical review and was exempted by the Johns Hopkins Internal Review Board being classified as non-human subjects research.

To supplement the interviews, firms’ publicly-available documentation on CSR was reviewed together with any non-public reports provided by respondents. This included but was not limited to: firm-provided CSR documents, annual reports, websites, published interviews and press releases. The most recent versions were utilized, usually from between 2010 to 2013; older sources were referred to when necessary. Triangulation of the interview responses, firm publications and secondary sources produced the results presented below.

An earlier draft of this manuscript was shared with all respondents to confirm information accuracy; appropriate edits were incorporated based on feedback.

### List of respondents’ titles

- Senior Manager, Corporate Responsibility
- Director, Public Engagement & Access Initiatives
- Vice President, Global Market Strategy
- Executive Director, Corporate Responsibility
- Communications Director, Sustainability and Access to Healthcare
- Lead, New Markets Division
- Head of Corporate Responsibility Strategy & Stakeholder Engagement

## Findings

Firms included in the study sample are GlaxoSmithKline, Janssen, Merck, Novartis, Pfizer and Roche. In presenting findings, we have randomly replaced firm name with a letter to preserve the anonymity of respondents and the firms they represent.

### CSR activities & organizational structure

CSR management is situated differently in each of the included firms. Although all interviewees participated directly in managing corporate social responsibility activities, job titles and departments varied. Table 1 represents ways in which each firm incorporated CSR into its organization.

**Table 1:** **Organization: situating CSR within the corporation**

	**A**	**B**	**C**	**D**	**E**	**F**
**CSR Policies**						
Clearly articulated definition of CSR/CR	X	X		X	X	
Clear Criteria for CR activities	X				X	
Specific Policies (internal or external) dictating CR			X		X	X
**Positioning Within Firm**						
CR department	X			X	X	
CR steering committee/group	X	X	X	X	X	X
Separate philanthropy entity (i.e. foundation)	X				X	
Sustainable business/shared value sector			X	X	X	
Systemic sustainable shared value business model			X			X
**Partnership**						
With competing firms	X	X	X	X	X	
With other private for-profit entities	X	X	X	X	X	X
With local governments	X	X	X	X	X	X
With local and international NGOs	X	X	X	X	X	X
Reporting*						
Independent	X	X	X	X		X
Integrated		X			X	X

*Refers to whether CR/CSR is incorporated into annual reports or a separate report is generated (or both).

Several of the firms have affiliated foundations that are separate legal entities from the corporation and are primarily responsible for philanthropic giving. Others include this philanthropy as part of their CSR. Firm E’s respondent explained the breadth of their approach:

*We view C[S]R as a spectrum ranging from philanthropy to non-profit activities, to shared value activities where we pursue initiatives that generate societal value and economic value for the company. —Respondent, Firm E*

Table 1 shows that firms C and F have systemically integrated CSR efforts across commercial operational divisions. This means that they do not have separate responsibility arms and instead aim to incorporate these values into all of their endeavors to form a business model that generates value for both the enterprise and communities it serves.

Pharmaceutical firms engage in a wide array of activities under the umbrella of CSR which is represented in Table 2. These aggregated activities are limited to those programs that were identified either in publicly available documents produced by the firms themselves or in the semi-structured interview. Across the world, all firms offered differential pricing schemes for their products, whereby the same product is sold at different prices according to a country’s economic viability. Product donations are central to pharmaceutical companies CSR; all but one company indicated provision of drugs at no cost to patients as part of their efforts. mHealth (mobile health) initiatives were also popular among the firms. An example of such an initiative is the development of SMS-based appointment reminders for patients needing provider-assisted treatments which helps with adherence and supports firms’ pharmacovigilance. Five of the firms worked directly with the private sector, which sometimes included informal healthcare providers. One example of this was Firm C’s program that provided loans and training to individuals looking to open accredited pharmacies in the urban slums of some of Africa’s largest cities. Almost all firms conducted specialized research to address diseases that exclusively or disproportionately affect developing nations. This was included as CSR since firms expect insignificant financial returns on products being developed for countries with negligible ability to pay.

**Table 2:** **CSR activities by pharmaceutical firm**

	**A**	**B**	**C**	**D**	**E**	**F**
**Product Involvement**						
Donation	X	X	X	X	X	
Differential pricing sales for resource poor countries	X	X	X	X	X	X
Special licensing agreements for resource-poor countries	X			X	X	X
**Health Systems Strengthening**						
Training of health care workers	X		X		X	X
Improved local manufacturing	X		X			X
Increased product distribution capacity	X	X	X	X	X	X
Infrastructure investment		X	X			X
Supply-chain support			X	X	X	X
Private or informal provider engagement	X	X	X		X	X
Promoting uptake of health insurance	X					X
**Miscellanous**						
Loan & Microfinance programs			X		X	X
mHealth initiatives		X	X	X	X	X
Social marketing					X	
Health issue awareness campaigns	X	X		X		X
Advocacy and policy	X					
Targeted Research & Development*	X	X	X	X	X	X
International employee placement program	X	X	X	X		

*Specifically to meet developing country health needs or products with no commercial potential.

According to the interviewees, CSR activities were always supported by partnerships involving local and international partners, which were prominently featured on the firms’ websites. Firms described partnerships with telecommunications companies for their mHealth initiatives, banks for micro-loan schemes to increase patient purchasing power, and community-based organizations to encourage local buy-in and participation.

The decision-making process behind initiating specific CSR activities varied across the included firms but was often determined by the presence of existing in-country networks, cooperative local government, alignment with company expertise, and the ability to make health impact in an area that was not over-saturated they could meaningfully contribute to. While no firm identified specific exclusion criteria concerning CSR activities, several cited instances where they had foregone or withdrawn from an opportunity. Firm A explains one of these circumstances:

*About 2 or 3 years ago we looked at world hunger. There was a lot of interesting marketing involved in hunger initiatives and so we kind of put our toe in the water, and I think we quickly realized that to make a big difference or any difference in that space required a huge investment and we would probably have to reallocate […] most of our philanthropic resources into that to make a difference. And as we looked at that, we said yes, it’s a perfectly worthy thing to do but is it the right fit for [us]? —Respondent, Firm A*

In this quote, Firm A’s respondent alludes to “making a difference”, which introduces the motivations that drive firms to engage in CSR in the first place.

### Motivations for CSR

Data presented in Table 3 show motivations for CSR engagement that were disclosed in the interviews with firm representatives. The categories were generated by the researchers based on responses following the completion of all interviews.

**Table 3:** **Motivations identified by firm representatives**

	**A**	**B**	**C**	**D**	**E**	**F**
**Reputational benefit**						
Perception of firm by consumers	X	X	X	X		
Perception of firm by potential funders				X		
Relationship and trust building with potential partners and stakeholders	X	X				X
Employee recruitment, satisfaction, engagement and innovation	X	X		X	X	
Ranking on Dow Jones Sustainability Index*				X	X	X
**Competetive advantage**						
Entering new markets	X		X	X	X	X
Expanding consumer base						X
Intelligence gathering on new markets		X				
Anticipated long-term financial gain	X		X	X		X
Increased cost-effectiveness of interventions and programs (in developing countries)			X			
Improved efficiencies					X	
Opportunity for innovation	X					X
Special access to local government officials and decision making				X		
**Philanthropy & health impact**						
Obligation as a health care company				X		X
Improved population health impact	X	X	X	X	X	X
Increasing patient access to necessary medications/health services	X	X	X	X	X	X

*and/or Access to Medicine Index, Fortune World’s Most Admired Companies.

Population health impact was included by every firm as a source of motivation, either in the interview or in publicly available documents. The respondent from Firm F stated that health was his firm’s *“greatest asset […] and the contract that we have is delivering what the patients need*,” positioning population health as central to the firm’s purpose and alluding to its social obligation.

In direct response to why they work in developing countries, Firm C’s respondent stated that investment dollars could go further in LMICs, making CSR efforts more cost-effective than they might be elsewhere; this respondent went on to cite Africa as the *“last frontier of growth for many multinational companies.”* Several respondents indicated that by helping to create healthier communities in developing countries, they were fueling economic prosperity, increasing local purchasing power, and thus opening doors to new markets for other pursuits by the same firm. Firm B’s respondent noted that CSR was a *“way to grow business in a global marketplace.”* The same firm acknowledged that CSR initiatives helped them gather market intelligence and learn about health systems to inform decision making with regards to a new market. In this case, CSR work preceded business presence and provided feedback that informed business strategies for expansion into this market. Others identified pre-commercialization CSR presence as a way of building trust with local governments, non-governmental organizations and consumers. This explains why new market entry considerations are intrinsically linked with external perceptions of a firm. Reputation was the most frequently and readily cited reason for CSR by all firms. All of the firms saw health, CSR, reputation and sustainability as interrelated. Firm A exemplifies this:

*From a commercial perspective, […] we feel very strongly that being socially responsible and engaging in activities that both advance our business objectives as well as social objectives really will help the company to be sustainable over the longer term. […] Engaged in the business [of] health, it really does relate to trust and you really live and die on how trusted you are by patients and customers. […] Demonstrating a strong commitment to C[S]R really helps to build trust with key stakeholders—with patients and that will speak to our long-term sustainability. –Respondent, Firm A*

Cited by 4 of the 6 firms, appealing to potential employees was a common source of motivation driving programs that directly involve employees in CSR activities. An employee exchange programs is one example of this engagement which can be described as corporate-sponsored volunteerism where in-house employees are placed with organizations in developing countries to help them with their organizational governance and development strategies. One respondent acknowledged the importance of recruitment and its ties to reputation, stating that his firm wants:

*[…] to attract good employees [who] care about the reputation of the company and whether the company is ‘doing good’ –Respondent, Firm D*

### Evolving understanding of CSR

Firms are at different points in their integration and implementation of CSR. Many of them noted that their firm’s internal positioning of—and external position on—CSR was “in transition.” One respondent acknowledged that his firm was slow on the uptake and was looking to competitors who were already “*doing responsibility well*” as exemplars.

Several respondents were determined that while creating economic value is essential to shareholders, CSR, philanthropy and CSV initiatives can all be mechanisms to benefit society simultaneous to commercial activities. Firm B has placed philanthropy under their larger CSR umbrella, noting that for healthcare companies the two come from similar motivations. In contrast, Firms A and D separated philanthropy and CSR indicating that philanthropy should be wholly benevolent whereas CSR seeks to create commercially sustainable models that can be mutually beneficial to the firms and the recipient societies. In Firm D, the respondent explained that CSR currently straddles three major divisions of the company: “*corporate contributions, CSR & sustainability, and global health industry,*” crossing from philanthropic to commercial and what lies between.

There was a clear lack of consensus on the definition of CSR. A handful of firms did define CSR, either in the interview or in official documents, each with different wording or even meaning. A few firms expressed frustration about misconceptions of CSR among stakeholders including implementing partners, governments, internal constituents and consumers. Firm F cited a lack of understanding concerning CSR as hindering the private sector’s ability to contribute to global health programmatic development in LMICs. Critics, Firm F purports, are contributing to this impediment:

*Every push-back I hear is simply from people not understanding what they’re talking about […]; inflammatory use of these terms is harming the value of the ideas. –Respondent, Firm F*

Several of the respondents identified the lack of key performance indicators, best practices, transparency and data as major challenges to achieving CSR objectives. Some but not all firms did report using guidelines developed by the Global Reporting Initiative, an international organization that promotes the use of CSR reporting as a way for organizations to become more sustainable and contribute to sustainable development.^b^ Firm E perceived the Dow Jones Sustainability Index,^c^ Access to Medicine Index^d^ and Fortune’s list of the World’s Most Admired Companies^e^ as measures of reputation, which are intangible and hard to quantify. Several other firms also pointed to these indices as important incentives for CSR engagement by facilitating benchmarking of such activities. Firm F stated the specific annual goal of being among the top 3 companies in the industry on the Dow Jones Sustainability Index as a way of measuring and providing internal incentives for different departments.

## Discussion

To the best of our knowledge, this paper provides the first portrait of why multinational pharmaceutical corporations are engaging in global health-related CSR activities. All six firms interviewed had extensive CSR experience encompassing diverse initiatives from mHealth to preferential drug pricing and employee exchange programs. An external assessment has shown that employee engagement in CSR activities increases employee pride and loyalty, improves other stakeholders’ perceptions of the firm, and increases efficiency and impact of the host organization [10]. Existing literature purports that responsible pharmaceutical firms will necessarily include diverse public-private and private-private partnerships in their CSR agenda [17,20], which was supported by this study’s sample. While internal governance of CSR was diverse across the firms, many of the motivations driving CSR were consistent.

Motivations offered by the firms can be generalized into three interrelated categories: reputational benefit, competitive advantage and philanthropic health impact*.* Increasing access to medicines and treatments as a means to improving population health was the most commonly cited motivation for CSR endeavors in LMICs. This push for increasing access was closely linked to reputational benefit for the respondents, which the firms in turn connected to competitive advantage. CSR literature echoes many of the motivations cited in this study, including factors such as reputation building, opportunities for entering new markets and moral arguments to protect and better society [12,17,21,22].

Other research has found that pharmaceutical firms are “beginning to realize that, in many cases, meeting some needs of the underserved in LMICs may prove an important source of future growth and profitability” [20]. This is affirmed by firm respondents’ indications that CSR strategies offer opportunities for exploring and developing new markets in LMICs while simultaneously creating value through health for the people of these countries.

This study showed that pharmaceutical firms struggle with how other actors perceive and define CSR and that CSR is not even understood in the same way across the pharmaceutical industry. In his analysis of pharmaceutical firms’ CSR, Leisinger identifies a “wide pluralism of values” in the industry, resulting in the multiplicity of CSR definitions [12]. The UN offers vague suggestions for different levels of CSR standards ranging from a minimum where “businesses fulfill their legal obligations or, if laws or enforcement are lacking, that they ‘do no harm;’” to a maximum, which is “the active alignment of internal business goals with externally set societal goals (those that support sustainable development)” [13].

While company respondents were aware and in some cases focused on the various indices tracking CSR, the lack of clarity around the definition of CSR can be problematic for these indices too. For example, the Access to Medicine Index has seven different dimensions which include philanthropy, quite separately from “general access to medicines.” However, as this study has demonstrated, the relationships between philanthropy and broader access to medicines strategies vary substantively across firms. The Dow Jones Sustainability Index is the best established of the various CSR-related indices discussed but its focus is more on corporate sustainability than public health concerns. The Access to Medicines Index provides a much stronger focus on public health, but as others have argued, has weaknesses including 30% of indicators being “inadequately specified,” and some key CSR dimensions (such as availability, access and affordability of essential medicines) either being omitted or inadequately weighted [23]. In order to improve transparency and accountability, a common definition of CSR needs to be adopted by pharmaceutical and healthcare companies, and the Access to Medicine index measures of CSR effort need to be further developed.

This study demonstrates that across the pharmaceutical industry, multinational corporations are making significant and diverse CSR efforts influencing health in LMICs. There are substantial further questions for research. First, while CSR initiatives are intended to deliver social benefits, it is possible that there may also be unforeseen consequences; for example, training certain health providers may squeeze others out of the labor market [24]. This study did not address the nature of CSR impacts on intended beneficiaries and research in this area would be worthwhile. Second, although pharmaceutical firms are building CSR initiatives on local partnerships, research is needed to investigate the extent to which such initiatives are truly aligned with local policies and priorities. Finally, our interviews and document review did not reveal data concerning the magnitude of resources currently invested in CSR and further investigation of the resources CSR requires is warranted.

### Limitations

This was a small-scale exploratory study constrained by the resources available to the researchers. Interviewees’ roles were not uniform across the firms and thus they may be responding from the particular perspective of their own position, rather than giving an overarching view of the firm’s CSR practice. Nonetheless, we believe that the combination of interviews and document review provided a relatively comprehensive view of types of CSR activities conducted, organizational structures for managing CSR, and motivations for CSR across the sample of firms. It should be noted that only established companies with relatively mature CSR strategies were included in this study, and smaller companies with more nascent CSR initiatives may offer different motivations and perspectives.

## Conclusions

This study highlights the increasingly important role that corporate social responsibility is playing in large pharmaceutical firms, and by extension in the health sectors of low- and middle-income countries. While the UN suggests that “CSR offers real opportunities for the governments of middle and low-income countries to change the terms on which they interact with business […] and to leverage additional resources through partnership” [13], the public health community is a long way from understanding how best to go about this. Furthermore, several of the factors unveiled in this study seem to limit the ability of the largest pharmaceutical companies to maximize their resources and will to improve the health of underserved populations.

Our study points to three key steps that should be taken to help move forward the dialogue between the CSR arms of large firms and people concerned about public health in low- and middle-income countries. First, there is a need for clearer definitions of the many terms that are currently bandied around in this field, including philanthropy, CSR and CSV. Pharmaceutical companies suggest that misunderstanding of these terms leads to doubts regarding their CSR efforts and motivations, but it is clear that confusion also exists within the industry. Second, existing indices to track the development, implementation and effects of CSR strategies in the pharmaceutical sector should be strengthened. Of the available indicators the Access to Medicines Index is best aligned with public health interests, but needs more work in terms of garnering attention, promoting transparency and ensuring that the indicators truly reflect existing priorities and concerns. Finally, as detailed in the discussion, this exploratory study has suggested multiple further lines of enquiry, most of which would require country level studies to investigate in a more detailed way how specific CSR initiatives have engaged with country health systems and the impacts they have made. It is clear that as CSR grows in significance the global health community would be well-advised to invest more in understanding this important practice.

### Endnotes

^a^Sources included firm-produced financial statements and rankings from the following websites: FiercePharma, Drug Watch, Forbes and PMLive.

^b^For more information see: https://www.globalreporting.org/information/sustainability-reporting/Pages/default.aspx.

^c^For more information see: http://www.sustainability-indices.com/.

^d^For more information see: http://www.accesstomedicineindex.org/what-index.

^e^For more information see: http://fortune.com/worlds-most-admired-companies/.

## Abbreviations

CSR: Corporate social responsibility
CSV: Creating Shared Value
LMICs: Low- and Middle-Income Countries
UN: United Nations
